# 非小细胞肺癌脑转移免疫治疗的相关肿瘤微环境基础

**DOI:** 10.3779/j.issn.1009-3419.2019.08.06

**Published:** 2019-08-20

**Authors:** 青 兰, 春霞 纪, 瑜 姚

**Affiliations:** 200040 上海，复旦大学神经外科研究所，复旦大学附属华山医院神经外科 Immunology Laboratory, Neurosurgical Institute of Fudan University; Department of Neurosurgery, Huashan Hospital, Fudan University, Shanghai 200040, China

**Keywords:** 肺肿瘤, 脑转移, 免疫治疗, 肿瘤微环境, Lung neoplasms, Brain metastases, Immunotherapy, Tumor microenvironment

## Abstract

脑是非小细胞肺癌(non-small cell lung cancer, NSCLC)最常见的远处转移部位，脑转移也是晚期肺癌致残致死的主要原因。近年来，小分子酪氨酸激酶抑制剂的应用和疗效奠定了驱动基因突变阳性的NSCLC脑转移的治疗基础。随着程序性死亡受体1(programmed cell death protein 1, PD-1)/程序性死亡受体配体1(programmed cell death protein ligand 1, PD-L1)抑制剂及相应联合疗法的不断发展，免疫治疗已成为驱动基因突变泛阴性的NSCLC脑转移患者的重要选择，相关生物标志物的价值也日益凸显。由于NSCLC脑转移肿瘤及其微环境的免疫病理特征具有一定的特殊性，本文旨在回顾相关研究进展，并为免疫治疗联合策略的探索与新型免疫疗法的开发提供参考。

## Abstract (en)

Brain is the most frequent site for distant metastases of non-small cell lung cancer (NSCLC). Brain metastasis (BM) is also the leading cause of disabilities and death in advanced NSCLC. In recent years, the application and effectiveness of small-molecule tyrosine kinase inhibitors has formed the basis for the treatment of NSCLC brain metastases with driver gene mutations. With the development of programmed cell death protein 1 (PD-1)/programmed cell death protein ligand 1 (PD-L1) inhibitors and relevant combination therapies, immunotherapy has become an important choice for non-classic oncogene addicted NSCLC BM patients. Also, the roles of relevant biomarkers are increasingly standing out. By reason of the particular immunopathological features of NSCLC brain metastases and its microenvironment, the aim of this review is to summarize relevant research progresses and provide more references for combination strategies of different therapeutic methods as well as the development of novel immunotherapies.

## 1 前言

脑转移瘤是极为常见的颅内肿瘤，以肺癌为主要来源。在NSCLC的疾病发展过程中，约半数的患者会发生同时(synchronous)或异时(metachronous)的中枢神经系统(central nervous system, CNS)播散^[1-3]^，此外亦存在表现为“脑先行”的类型。目前，酪氨酸激酶抑制剂(tyrosine kinase inhibitors, TKIs)已成为驱动基因阳性NSCLC的一线治疗手段，其他传统治疗方法的角色亦得到了进一步的拓展。由于NSCLC脑转移患者的生活质量和生存时间显著受损，如何通过多学科手段应对这一难题始终是肺癌领域的研究热点之一。

近年来，以程序性死亡受体1(programmed cell death protein 1, PD-1)/程序性死亡受体配体1(programmed cell death protein ligand 1, PD-L1)抑制剂为代表的I-O(immuno-oncology)治疗异军突起，一系列临床试验确证了免疫检查点抑制剂(immune checkpoint inhibitors, ICIs)对NSCLC的治疗效果^[4-6]^，目前亦有小规模的临床研究提示免疫治疗可为部分驱动基因突变泛阴性的NSCLC脑转移患者带来福音，其中PD-L1单抗atezolizumab的作用较为突出^[7, 8]^。同时，免疫治疗联合放、化疗和抗血管生成药物等方案的相关研究也将对NSCLC及其脑转移的治疗策略产生重要影响。虽然关于NSCLC的基础和临床研究是肿瘤学最为前沿的领域之一，但由于颅内组织取材困难和形成脑转移的生物学机制较复杂等原因，研究者在进一步探索基于CNS免疫微环境的NSCLC脑转移治疗策略时仍需面对诸多挑战。在现代肿瘤病理技术的基础上，此领域的研究成果将为NSCLC脑转移免疫治疗生物标志物的选择、优化特定人群的免疫治疗策略以及新型免疫疗法的开发铺平道路。

## 2 全身应用ICIs的肿瘤免疫微环境基础

PD-1通路的活化是肿瘤细胞及其微环境中的免疫细胞削弱效应T细胞杀伤功能的常见机制，是肿瘤免疫逃逸的重要途径之一^[9, 10]^。由于PD-L1(即B7-H1)分子较广泛地表达于包括NSCLC在内的不同类型的肿瘤中^[11, 12]^，且基于肿瘤免疫正常化原理的PD-1通路阻断策略具有较高的安全性和有效性，故当前的ICIs主要为直接靶向于PD-1/PD-L1的抗体药物。虽然食品药品监督管理局(Food and Drug Administration, FDA)已批准将基于免疫组化(immunohistochemistry, IHC)的肿瘤细胞(tumor cells, TCs)和浸润性免疫细胞(immune cells, ICs)的PD-L1表达丰度检测作为筛选NSCLC免疫治疗优势人群的相关靶标，但由于其在肿瘤微环境中的表达具有不同的机制和一定的异质性，且可能受到既往治疗的影响，故仍存在部分可获益于上述治疗的PD-L1阴性患者，提示需结合其他指标^[13, 14]^。另一方面，基于二代测序技术(next-generation sequencing, NGS)的肿瘤突变负荷(tumor mutation burden, TMB)检测已被证实对于预测NSCLC的免疫治疗效果具有重要价值，并已被纳入相应美国国家综合癌症网络(National Comprehensive Cancer Network, NCCN)指南。TMB按样本来源可分为组织TMB(tissue TMB, tTMB)和基于循环肿瘤DNA(circulating tumor DNA, ctDNA)的血液TMB(blood TMB, bTMB)，一般以非同义突变(导致氨基酸改变的核苷酸变异)的总数量或每1 Mb(1兆碱基)的突变数量来表示，但其中只有小部分非同义突变可产生能被免疫系统识别并结合的突变肽段，且仍有部分高TMB的肺腺癌患者无法对免疫治疗产生响应，故将其作为单一的免疫治疗生物标志物亦具有一定的局限性^[15, 16]^。

与其他部位的转移瘤相似，NSCLC脑转移与其原发灶之间常存在肿瘤免疫特征上的时空异质性。Mansfield等研究者^[17]^对73例肺癌脑转移及其配对原发灶的病理标本进行了分析，发现在所有样本中PD-L1阳性表达者共占39%，小部分病例仅脑转移灶可见PD-L1的表达; 配对样本间肿瘤细胞PD-L1表达的不一致率为14%，而免疫细胞PD-L1表达的不一致率为26%，提示需考虑颅内外病灶间异质性的存在。此外，相当一部分颅内病灶与其原发灶相比存在着PD-L1表达或TILs浸润的丢失，在结合TILs浸润丰度和PD-L1表达水平^[18]^对配对样本的免疫微环境类型进行划分时，存在更多的脑转移瘤属于“免疫忽视”(或称“免疫无反应型”)状态，即PD-L1表达和TILs浸润均呈阴性。研究者后续对20例肺腺癌(lung adenocarcinoma, LUAD)脑转移及其配对原发灶的T细胞抗原受体β链互补决定区3(T-cell receptor β complementarity determining region 3)和TMB进行了检测^[19]^，发现颅内病灶与其原发灶相比有着相对更少的T细胞克隆数和更高的优势T细胞克隆丰度，且配对样本间T细胞克隆的重叠度有限，提示LUAD脑转移灶中T细胞克隆的丰富程度较原发肿瘤有所紧缩。从另一维度来看，13例LUAD脑转移瘤和原发灶的TMB平均值分别为24.9/Mb和12.5/Mb，提示颅内病灶总体存在相对较高的非同义肿瘤突变负荷，对免疫治疗或可产生一定的响应。

与前述研究有所不同的是，Zhou等^[20]^发现在25对原发NSCLC及其脑转移灶的配对样本中，颅内病灶TCs上的PD-L1表达水平总体高于其配对的原发灶，且同时性(在首次病理确诊NSCLC后的1个月内经影像诊断为脑转移^[21]^)配对样本中肿瘤细胞PD-L1表达水平的异质性要强于异时性的配对标本。该研究还提示了脑转移瘤实质内CD8^+^ TILs的密度总体上较其原发灶更低，且基质CD8^+^ TILs数量少的病例总体上具有更差的预后。此外，Kim等^[22]^探究了NSCLC脑转移患者颅内外病灶对PD-1单抗nivolumab或pembrolizumab的响应情况，并分析了另一队列中原发肺癌及其脑转移灶病理标本的免疫微环境特征。研究者对接受ICIs干预的队列进行疗效评价(RECIST 1.1标准)发现18例肺癌患者中有7例的颅内外病灶皆出现了疾病进展，而11例原发灶呈部分缓解或稳定状态的患者中有8例表现为颅内进展，提示部分脑转移瘤对免疫治疗可能表现为相对较差的响应; 另一队列的手术切除样本则提示了脑转移瘤中PD-1^+^ TILs的数量较原发灶更少，且在LUAD病例中，更多的CD3^+^ TILs和PD-1^+^ TILs浸润与更差的预后有关。

除PD-L1表达水平和TMB外，宿主体内一些其他的生物标志物亦被证实可有效预测免疫治疗的疗效。例如因缺失由人类错配修复(DNA mismatch repair, MMR)基因编码的任一蛋白所造成的DNA错配修复功能缺陷可导致微卫星不稳定(microsatellite instability, MSI)的发生，基于不同检测方法的微卫星高度不稳定(microsatellite instability-high, MSI-H)和错配修复功能缺陷(different mismatch repair, dMMR)亦为FDA批准的泛瘤种免疫治疗生物标志物。具备该特征的实体瘤常具有较强的免疫原性和广泛的T细胞浸润，因而对ICIs治疗有着良好响应，但此类患者在NSCLC人群中所占比例十分有限，故该指标目前在结直肠癌等其他瘤种中应用较广^[23, 24]^。此外，一些研究表明肠道微生物及其代谢产物不仅能影响其他部位肿瘤的免疫微环境和I-O治疗的效果^[25]^，甚至在动物模型中可作用于大脑的免疫细胞并调节相关疾病^[26]^。因此，一些新型的免疫治疗标志物对脑转移瘤微环境和免疫治疗的意义亦有待探究。

对脑转移瘤而言，血-脑屏障(blood-brain barrier, BBB)和血-肿瘤屏障(blood-tumor barrier, BTB)曾被认为是阻碍大分子药物发挥疗效的关卡，但已有研究^[7, 8]^证实全身应用ICIs对于防治NSCLC脑转移亦可发挥有效作用，其原因可能是部分药物能促进效应免疫细胞向颅内病灶的募集以及瘤区相关屏障的结构异常。在此背景下，免疫治疗能在何种程度上使伴有脑转移的NSCLC患者获益以及相应生物标志物的选择已成为许多研究者关注的焦点，同时还需考虑不同检测方法、判读标准和阈值划分等因素所造成的结果差异。值得关注的是，颅内外病灶可能对全身性的免疫治疗产生不同水平的响应，且有研究提示在接受免疫治疗前存在两个以上转移灶的NSCLC患者发生超进展(hyperprogressive disease, HPD)的概率明显较高^[27]^，已知MDM2/MDM4扩增和*EGFR*突变或可作为相关标志物^[28]^，但该现象与脑转移瘤的关系及潜在机制仍有待探究，同时在临床实践中还需考虑是否会出现假性进展和不表现为肿瘤缓解的临床获益等情况。此外，由于相当一部分脑转移瘤微环境中的效应免疫细胞存在着“质”和“量”上的缺失，通过不同策略解决这一问题必将有助于增强ICIs的疗效并扩大免疫治疗的优势人群。

## 3 相关研究对免疫治疗联合策略的启示

目前，手术治疗仍是缓解颅内孤立病灶所引起CNS症状的主要方法，而随着靶向药物种类的拓展和IO时代的来临，无症状NSCLC脑转移的治疗格局已发生深刻变革。另一方面，立体定向放射外科(stereotactic radiosurgery, SRS)和全脑放射治疗(whole brain radiotherapy, WBRT)在有症状和多发脑转移的治疗中始终扮演着重要角色，目前也有许多放疗联合免疫治疗的临床试验正在进行。就局部作用而言，放疗可通过直接杀伤肿瘤细胞使更多的肿瘤相关抗原(tumor-associated antigens, TAAs)得以暴露，还可增加微环境中多种促炎细胞因子的释放，从而促进CD8^+^ T细胞向肿瘤微环境中的浸润，协同ICIs发挥作用。同时，放疗引起的TGF-β的活化可通过调节CD8^+^ T细胞的克隆扩增和细胞毒作用、抑制CD4^+^ T细胞的分化和影响肿瘤相关性巨噬细胞(tumor-associated macrophages, TAMs)及中性粒细胞等细胞的功能促进抑制性免疫微环境的形成^[29]^。此外，已有基于小鼠胶质母细胞瘤(glioblastoma multiforme, GBM)模型的研究^[30]^表明放疗的延迟效应可致使促进胶质母细胞瘤侵袭生长的脑微环境的产生，但该作用对脑转移瘤的影响尚待探究。从远隔效应(abscopal effect)的角度来看，TAAs特异的T细胞的迁徙可增强具有相同抗原的其他病灶的抗肿瘤免疫反应，多病灶放疗或将更有效地促进这一作用^[31]^。此外亦有回顾性研究^[32]^提示对于原发灶呈PD-L1阴性表达的NSCLC患者，放疗可能导致脑转移灶出现PD-L1表达的阳性转变。综上，不同形式的放疗可在不同时间点通过多种途径影响免疫治疗，其对于NSCLC脑转移肿瘤细胞及其免疫微环境的作用仍需通过更多的研究予以证实。

化疗亦为NSCLC脑转移的传统治疗手段之一，尤其是在对原发灶具有明显疗效的情况下。与放疗类似，化疗通常会减少血液循环中淋巴细胞的数量，使得机体的免疫功能受到一定程度的抑制。但其在减轻肿瘤负荷的同时或可使更广范围的TAAs得以暴露，还可部分激活树突状细胞(dendritic cells, DCs)等抗原呈递细胞(antigen-presenting cells, APCs)，增强局部TAAs的交叉提呈，从而促进效应T细胞的作用并诱导记忆T细胞的形成^[33]^。此外亦有基于多中心样本的回顾性研究^[34]^提示全身性化疗对NSCLC脑转移瘤PD-1/PD-L1的表达和ICs的浸润均未见明显影响。因此，化疗联合免疫治疗的策略可能对于NSCLC转移瘤的治疗具有一定的应用价值^[35]^，但具体方案的选择及其作用亦有待进一步的研究。

近年来，以贝伐珠单抗(bevacizumab)为代表的抗血管生成药物在NSCLC脑转移治疗中的安全性和有效性已获公认。有研究^[36]^分析了NSCLC脑转移样本及其配对原发灶中VEGF和CA9的表达以及微血管密度和血管成熟度的差异，提示脑转移瘤中的成熟血管比例更高，或可导致其对于抗血管生成药物的反应有所不同。已知VEGF可通过抑制APCs的成熟和抗原呈递、阻碍效应T细胞的迁徙和杀伤功能、促进抑制性ICs的募集等方式负向作用于机体局部和系统的抗肿瘤免疫反应，促进肿瘤的增殖和转移，故相应抗体或可在促进肿瘤血管正常化的同时重塑肿瘤微环境并增强ICIs的作用^[37, 38]^。目前，抗血管生成药物联合免疫治疗已在晚期NSCLC患者中表现出良好的效果^[39]^，亦有相应的针对脑转移的临床试验正在进行。

还需注意的是，糖皮质激素的使用对于有症状的肺癌脑转移患者十分常见，但在接受免疫治疗的转移性NSCLC人群中，早期应用糖皮质激素与不良的疾病控制和较短的生存时间相关^[40]^。有研究^[34]^提示NSCLC脑转移瘤术前对糖皮质激素的使用可减少ICs上的PD-L1表达水平，但总体上不影响ICs向肿瘤组织的浸润。就联合免疫疗法而言，细胞毒性T淋巴细胞抗原4(cytotoxic T-lymphocyte-associated protein 4, CTLA-4)抑制剂或可增加T细胞向肿瘤区域的募集，从而提高PD-1通路抑制剂的疗效^[41]^。此外，吲哚胺-2, 3-双加氧酶(indoleamine-pyrrole 2, 3-dioxygenase, IDO)抑制剂亦为具有一定前景的抗肿瘤药物，其可阻断IDO-1酶在淋巴细胞色氨基酸代谢过程中的作用从而修复机体的抗肿瘤免疫，或可在将来为更多患者带来福音。已有研究证实IDO在NSCLC中存在着相当广泛的表达^[42]^，目前亦有将相应联合疗法应用于脑转移人群的临床试验正在进行。另一方面，对于驱动基因突变阳性的患者，免疫治疗联合TKIs的疗效常与具体的突变类别有关。已知NSCLC脑转移常伴有*EGFR*突变^[43]^，但*EGFR*突变的肺癌病灶多表现为抑制性的肿瘤免疫微环境^[44]^，且靶向药物联合免疫治疗可能更易引起相关毒性反应^[45]^。目前，免疫治疗对于TKIs耐药的肺癌脑转移人群的意义仍有待进一步明确。

综上，不同联合策略对NSCLC脑转移瘤微环境和免疫治疗疗效的作用还需更多的回顾性和前瞻性研究予以说明，同时还需探究相应疗法的种类、剂量和时间窗可能带来的影响。

## 4 免疫治疗新靶点的开发与微环境中其他组分的作用

虽然CNS曾被认为是体内存在“免疫豁免”的区域，但已有研究证实其与外周淋巴结间存在独特的淋巴引流系统，且可能对颅内肿瘤的发生发展具有一定的影响^[46]^。根据免疫治疗的作用机制，效应T细胞向肿瘤微环境中的迁移和浸润是抗肿瘤免疫反应的重要环节，且与对ICIs的治疗反应有关^[47]^。近期研究^[48]^发现在多种颅内肿瘤中，幼稚T细胞上S1P1的丢失可引起T细胞在骨髓中的扣留(sequestration)，这一T细胞功能障碍的新模式或许是导致抗原忽视的重要原因。此外，调节性T细胞(regulatory T cells, Tregs)亦可通过对S1P1受体的作用促使CD8^+^效应T细胞囚禁于肿瘤引流淋巴结(tumor-draining lymph node, TDLNs)内并显著抑制杀伤性T细胞抗肿瘤效应^[49]^。对于包括部分NSCLC脑转移瘤在内的微环境呈TILs低水平浸润的颅内肿瘤，逆转上述过程可能对增强ICIs和各类新型细胞治疗的作用效果具有重要意义。

类似于PD-L1(B7-H1)，B7-H4(B7S1, B7x)亦为B7分子家族中的一员，可促进肿瘤细胞的免疫逃逸^[50]^且在较高级别脑胶质瘤和NSCLC中有一定量的表达^[51, 52]^。Li等^[53]^发现在49例NSCLC脑转移中有40.8%的样本存在B7-H4的高表达，且颅内病灶中该分子的表达具有高于原发肿瘤的趋势。此外，原发肺癌高表达B7-H4的患者具有更高的发生脑转移的风险，且B7-H4的表达与更差的预后相关。因此，B7-H4可能是部分NSCLC及其脑转移患者潜在的免疫治疗靶点，基于IHC的B7-H4表达评分或可作为相应的生物标志物。

**1:** 正在开展的NSCLC脑转移ICIs联合疗法的临床试验 Summary of ongoing trials combining ICIs and other therapies for patients with NSCLC brain metastases

Category	NCT Identifier	Drug arms	Phase
ICI+CT	03526900	atezolizumab+carboplatin+pemetrexed	Ⅱ
ICIs+RT	02978404	nivolumab+SRS (15 Gy-20 Gy in 1 fraction)	Ⅱ
	02858869	pembrolizumab+SRS (6 Gy *vs* 9 Gy *vs* 18 Gy-21 Gy)	Ⅰ
	02696993	nivolumab+SRS *vs* nivolumab+WBRT *vs* nivolumab+ipilimumab+SRS *vs* nivolumab+ipilimumab+WBRT	Ⅰ, Ⅱ
ICI+anti-VEGF antibody	02681549	pembrolizumab+bevacizumab	Ⅱ
ICI+anti-IDO-1 agent	03343613	LY3381916 (anti-IDO-1 agent)+LY3300054 (anti-PD-L1 antibody)	Ⅰ
ICIs: immune checkpoint inhibitors; NSCLC: non-small cell lung cancer; CT: chemotherapy; RT: radiation therapy; SRS: stereotactic radiosurgery; WBRT: whole brain radiation therapy; IDO: indoleamine-pyrrole 2, 3-dioxygenase; PD-L1: programmed cell death protein ligand-1.			

除外多种重要的抑制性免疫分子，宿主脑微环境^[54]^和外周血中的多种免疫细胞亦在NSCLC脑转移瘤的发生发展中扮演着重要的角色。Teglasi和Reiniger等^[34]^在208例LUAD脑转移样本中首次证实了约半数的病例存在瘤周的单核细胞浸润(即单核细胞环)，且与较高的基质ICs数量和更好的预后相关。其中驻留于CNS内的小胶质/巨噬细胞是对转移瘤产生免疫反应的主力军之一，其在微环境中不同因素的刺激下可极化为以分泌促炎细胞因子、iNOS高表达和增强T细胞抗肿瘤效应为特征的M1表型，或是促进新生血管形成和抑制抗肿瘤免疫的M2表型^[55, 56]^。体外实验^[57]^证实小胶质/巨噬细胞在肿瘤脑转移早期的免疫炎症效应中发挥着重要作用，且有研究^[58]^发现在肺癌脑转移病灶周围，表达Iba-1的小胶质细胞的数量明显增加，并在肿瘤与正常脑组织间围绕形成明显边界。然而，转移瘤部位只有少数小胶质细胞表达iNOS和TNF-α，表明这些小胶质细胞发生了不同模式的活化。星形胶质细胞(astrocytes)亦是CNS中具有代谢支持功能的重要组分，且可参与脑转移瘤放射性坏死灶周围炎症反应的形成^[59]^。研究^[58]^表明NSCLC脑转移瘤周围存在GFAP阳性的星形胶质细胞的增生，且其中的小部分可表达TNF-α。亦有研究^[60]^发现人和小鼠的肿瘤细胞转移至脑部后表现为PTEN的表达丢失，且离开脑部微环境后，转移瘤细胞的PTEN表达得以恢复。这一依赖脑部微环境的、可逆的转移瘤PTEN转录和表达的下调是由起源于星形胶质细胞的外泌体中的相关microRNA在表观遗传水平进行调控的。脑转移瘤中这一适应性的PTEN丢失可经相关通路增加趋化因子CCL2的分泌，从而募集Iba-1阳性的小胶质/巨噬细胞，促进转移瘤的增殖并抑制其凋亡。因此，靶向于抗炎表型的小胶质细胞群和抑制星形胶质细胞的相关作用可能是治疗脑转移瘤的有效手段。

Th17细胞亦为肿瘤微环境中常见浸润的免疫细胞类型，可分泌IL-17等多种细胞因子并通过CXCR2途径诱导NSCLC肿瘤新生血管的形成，但该类细胞在肺癌等肿瘤发生发展过程中的作用尚无定论。有研究^[61]^表明在伴有脑转移的NSCLC患者的外周血中，Th17细胞数量和血浆IL-17的水平均高于正常人。另一方面，肺癌脑转移患者脑脊液中IL-17的水平高于无脑转移的肺癌患者，提示Th17细胞和IL-17参与了肺癌发生脑转移的机制。因此，Th17和IL-17对NSCLC脑转移的意义有待更进一步的研究，并可能成为潜在的免疫治疗靶点。此外，特定的趋化因子和趋化因子受体如CXCL12/CXCR4轴已被证实在肿瘤的侵袭和转移过程中发挥着重要作用，有基于M0期和M1期NSCLC匹配样本的研究^[62]^发现CXCL12和CXCR4在脑转移瘤中存在着免疫活性的增加，故该通路的阻断可能对转移瘤的防治具有积极作用。同理，血液循环和肿瘤微环境中其他类型免疫细胞和细胞因子在NSCLC脑转移瘤中的存在情况及作用亦有待更多的研究予以阐明。

## 5 细胞疗法的前景与展望

近年来，全外显子测序技术(whole exome sequencing, WES)的应用可帮助研究者挖掘肿瘤的特异突变，通过计算机模拟计算出TILs可能识别的突变抗原并应用于相关的过继性免疫细胞治疗^[63, 64]^;评价树突状细胞疫苗(dendritic cell vaccine, DCV)用于治疗NSCLC脑转移安全性和有效性的临床试验也即将开展。除不同类型的肿瘤疫苗外，以嵌合抗原受体T细胞(chimeric antigen receptor T cells, CAR-T)疗法和T细胞受体基因工程T细胞(T-cell receptor engineered T cells, TCR-T)疗法为代表的新型免疫细胞疗法已在实体瘤治疗领域取得了一定突破，目前亦有针对晚期NSCLC人群的相应研究^[65]^，如抗NY-ESO-1鼠TCR转导的自体外周血淋巴细胞(anti-NY ESO-1 murine T-cell receptor-transduced autologous peripheral blood lymphocytes)疗法联合化疗应用于脑转移瘤的治疗。值得注意的是，直接将体外培养扩增的免疫细胞靶向输注至脑部病灶的技术亦可能解决部分脑转移患者免疫细胞浸润缺失的问题，并有助于增强上述疗法的成效。

随着肿瘤免疫治疗向着“精准、联合、多样化”的趋势不断发展，NSCLC脑转移患者的生活质量与生存时间将得到显著的提升。在不断深入了解肿瘤与其免疫微环境协同进化过程的基础上，现有的回顾性研究成果或可为更多的前瞻性研究进一步指明解决问题的方向。此外，功能成像和分子影像技术的进步亦为免疫治疗领域带来了新的契机，其中免疫PET技术(immuno-PET)的优势尤为突出^[66, 67]^。该方法以正电子发射体标记特定探针并输入体内与相应靶点结合，可通过相应设备达成对肿瘤的“全面、无创、实时的免疫组化染色”或追踪特定免疫细胞，从而协助医生充分了解机体对免疫治疗可能产生的反应，并使患者实现更大的临床获益。此外，以脑脊液和外周血等样本为载体的液体活检亦可在一定程度上反映NSCLC脑转移和脑膜转移的表型特征和基因背景^[68]^，且具有微创和简便等优点，同样具有广阔的研究前景。

综上，针对NSCLC脑转移瘤免疫治疗的基础和临床研究已取得许多突破，如何通过多学科手段进一步推动相关领域的进展仍是值得关注的热点问题。
